# Zebrafish Models of Autosomal Dominant Ataxias

**DOI:** 10.3390/cells10020421

**Published:** 2021-02-17

**Authors:** Ana Quelle-Regaldie, Daniel Sobrido-Cameán, Antón Barreiro-Iglesias, María Jesús Sobrido, Laura Sánchez

**Affiliations:** 1 Department of Zoology, Genetics and Physical Anthropology, Faculty of Veterinary Science, Universidade of Santiago de Compostela, 27002 Lugo, Spain; ana.quelle@usc.es (A.Q.-R.); lauraelena.sanchez@usc.es (L.S.); 2 Department of Functional Biology, CIBUS, Faculty of Biology, Universidade de Santiago de Compostela, 15782 Santiago de Compostela, Spain; ds918@cam.ac.uk; 3 Instituto de Investigación Biomédica de A Coruña (INIBIC), Servicio Galego de Saúde, 15006 Coruña, Spain; ssobrido@gmail.com; 4 Preclinical Animal Models Group, Health Research Institute of Santiago de Compostela (IDIS), 15706 Santiago de Compostela, Spain

**Keywords:** zebrafish, hereditary dominant ataxias, spinocerebellar ataxias, expanded repeats, X-fragile, neurodegenerative disorders, genetic edition

## Abstract

Hereditary dominant ataxias are a heterogeneous group of neurodegenerative conditions causing cerebellar dysfunction and characterized by progressive motor incoordination. Despite many efforts put into the study of these diseases, there are no effective treatments yet. Zebrafish models are widely used to characterize neuronal disorders due to its conserved vertebrate genetics that easily support genetic edition and their optic transparency that allows observing the intact CNS and its connections. In addition, its small size and external fertilization help to develop high throughput assays of candidate drugs. Here, we discuss the contributions of zebrafish models to the study of dominant ataxias defining phenotypes, genetic function, behavior and possible treatments. In addition, we review the zebrafish models created for X-linked repeat expansion diseases X-fragile/fragile-X tremor ataxia. Most of the models reviewed here presented neuronal damage and locomotor deficits. However, there is a generalized lack of zebrafish adult heterozygous models and there are no knock-in zebrafish models available for these diseases. The models created for dominant ataxias helped to elucidate gene function and mechanisms that cause neuronal damage. In the future, the application of new genetic edition techniques would help to develop more accurate zebrafish models of dominant ataxias.

## 1. Introduction

Hereditary ataxias are a heterogeneous group of neurodegenerative disorders with clinical and genetic variability caused by dysfunction of the cerebellum and its afferent and efferent connections, mainly involving the cerebellar cortex, the dentate nuclei and the nuclei of the lower olive. In addition, degeneration of the Purkinje cells is a common feature because they may be more susceptible to genetic or functional insults than other neuronal cell types [1]. Ataxias are mainly characterized by slowly progressive incoordination of gait and often associated with poor coordination of hands, speech, and eye movements [2,3].

Clinical classification was historically difficult due to similar neuropathology clinical phenotype overlap. The development of molecular genetics allowed more accurate classification of ataxias based on their genetics [4]. The inheritance of ataxias can be autosomal dominant, autosomal recessive, X-linked or through maternal inheritance as part of a mitochondrial genetic syndrome [2].

Autosomal dominant spinocerebellar ataxias (ADCAs) are late-onset disorders restricted to the cerebellum and its connections, characterized by progressive gait and limb ataxia, variably associated with other non-neuronal and neurological symptoms such as peripheral neuropathy, ophthalmoplegia, retinopathy, pyramidal and extrapyramidal signs, dementia, and epilepsy [5,6]. ADCAs can be divided into spinocerebellar ataxias (SCAs), of which 43 loci have been described at the present time, and episodic ataxias (EA), which are seven different illnesses that can be distinguished by recurrent episodes of vertigo and ataxia, variably associated with progressive ataxia [7,8]. In addition, there are other conditions that have ataxia as a symptom that we will not discuss in this review.

Ataxia dominant related proteins have a wide range of functions including ion transport, deubiquitination, phosphorylation, dephosphorylation, and regulation of transcription and translation [6]. Alteration of their molecular pathways can cause neurotoxicity as a consequence of protein aggregation, ARN toxicity, alterations in calcium homeostasis, impaired proteostasis, mitochondrial stress, autophagy, apoptosis or deficits at the DNA/RNA level causing problems in DNA repair or transcriptional dysregulation [3,7].

**Figure 1:**
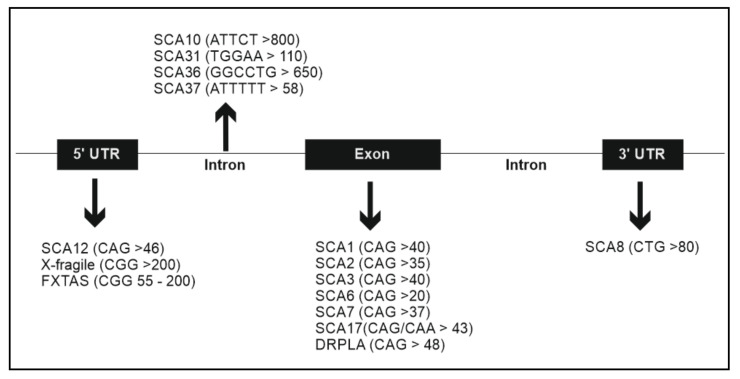
Repeat expansions in different ataxias.

Pathology of autosomal dominant ataxias is caused by genetic mutations that can be: polyglutamine expansions (e.g., CAG repeats), non-coding repeat expansions and conventional mutations: missense, insertions and deletions [1]. Pathological expansion is a common cause in dominant ataxias. Expansion diseases arise from normal polymorphic repeats in the population that moved into the pathogenic range by de novo or hereditary mutations. These deleterious expansions are believed to result in gain of function and the length of the repeat expansion is related with the age of onset and disease severity. Severity is increased in subsequent generations of a family in a process called anticipation Polyglutamine expansions promote misfolding of the disease proteins and altered DNA-protein or protein–protein interactions, which can cause transcription dysregulation, inhibition of the function of histone acetyltransferases and the formation of cytoplasmic or intranuclear neuronal polyglutamine aggregates. Non-coding repeat expansions are thought to cause gain of function by RNA toxicity, disrupt splicing of essential genes and can be subjected to RAN translation, resulting in the production of toxic protein species [1,9] (Figure 1). Repetitive elements have not been very widely studied in fish, although it is known that there are microsatellites in coding and noncoding positions but not if they have a role in pathogenesis [10].

As there are no effective treatments for dominant ataxias, animal models are useful for gaining insight into the molecular and cellular mechanisms that cause neurodegenerative disorders and helping to achieve the final goal of developing candidate therapies [11]. Despite all the research conducted with human cell models, these have the limitation of lacking the complex nervous connections that are present in a complete central nervous system (CNS) [12].

**Figure 2:**
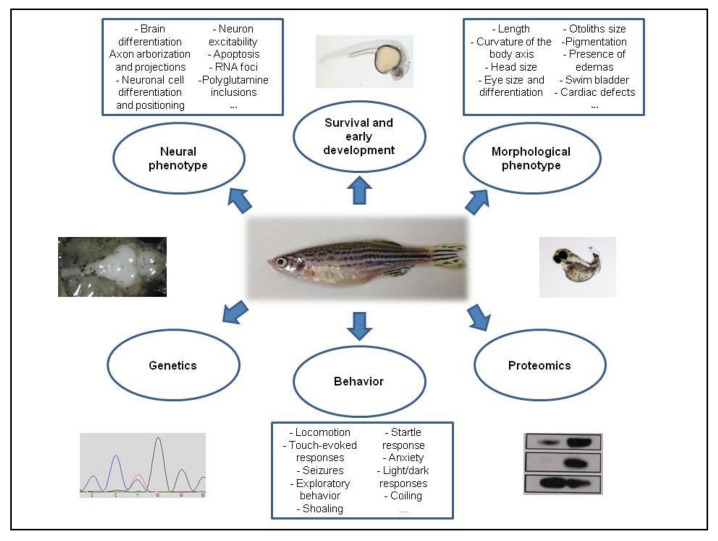
Summary of the main tests that can be applied to zebrafish models of neuronal diseases. Dorsal view of the brain to the left, female adult zebrafish in the center and a zebrafish embryo with morphological defects to the right.

Zebrafish (*Danio rerio*) is an important model for biomedical research that presents many advantages such as external fertilization, optic transparency of the embryos and larvae and 76 to 82% of genes related with human disorders are present in zebrafish [13,14]. The use of zebrafish in the study of neurodegenerative disorders is gaining popularity because it has a similar general organization of CNS to humans, presenting molecular and structural homology of the main brain areas and with most of its neuronal genes showing similar functions [15]. In addition, its behavioral patterns are easy to analyze, allowing the modeling of movement disorders, and it is useful for high-throughput screening of chemicals/drugs because embryos/larvae easily absorb compounds through the skin [16] (Figure 2).

The development of zebrafish loss of function models is easily achieved by knock-down with antisense morpholinos, which have a transient effect that remains for less than a week [17,18], or stable knocks out with mutagens like N-ethyl-N-nitrosourea (ENU) [19] or the recently developed CRISPR/Cas9 techniques [20]. Generation of gain of function models is more difficult, because knock-in methods are less effective in zebrafish [21]. However, temporal gain of function models are easily created by the overexpression of mRNAs [22].

Here, we used the classification of Bird [8] of dominant ataxias and dominant inherited disorders related with ataxia to search in the zfin database (https://zfin.org/) and in the PubMed database (https://pubmed.ncbi.nlm.nih.gov/) for potential zebrafish models developed for these genes and corresponding disorders to review the most important discoveries. We discuss the models of spinocerebellar ataxias (developed for SCA2, SCA3, SCA6/episodic ataxia 2, SCA7, SCA13, SCA14, SCA17 and SCA37) and other ataxias (sensory dominant ataxia 1, episodic ataxia 1 and episodic ataxia 5). We also review the zebrafish models created for X-fragile/fragile-X tremor ataxia syndrome because, although it is an x-linked ataxia, it is a gain of function disorder produced by repeat expansions. The most relevant data of the ataxias discussed here are summarized in Table 1. The zebrafish models reviewed here are summarized in Supplementary Table S1 (Table S1).

## 2. Materials and Methods

Articles were found in the zfin database (https://zfin.org/) by searching for zebrafish models of genes that cause dominant ataxias and dominant inherited disorders related with ataxia as described in the classification of Bird [8] and zebrafish models created for fmr1 gene (mutations on these gene causing X-fragile and fragile-X tremor ataxia syndrome).

The PubMed database (https://pubmed.ncbi.nlm.nih.gov/) was used to find additional models related to these diseases (especially those created by overexpression of repeated expansions) searching for “zebrafish” and “name of the disease”. The articles included in this review were published before 15 January 2021.

## 3. Results

### 3.1. Spinocerebellar Ataxias

#### 3.1.1. SCA2

Spinocerebellar ataxia type 2 is caused by CAG expansions in the gene ataxin-2 (*ATXN2*), which is an RNA-binding protein that interacts with the poly (A)-binding protein and regulates mRNA stability [23,24,25,26].

Chantal Sellier and colleagues [48] generated the first zebrafish model related to SCA2 while they were studying the *c9orf72* gene, a gene responsible of amyotrophic lateral sclerosis and frontotemporal dementia (ALS-FTD). First, they produced a knock-down of *c9orf72,* which led to inhibition of autophagy and accumulation of cytoplasmic aggregates of *p62/sqstm1* and *tdp-43*. These are histopathological characteristics of ALS-FTD patients. The combination of the *c9orf72* knock-down with the expression of a HA-tagged construction of intermediate *atxn2* polyglutamine expansions (30 repeats), potentiated the aggregation and neuronal cell death of *atxn2*. Zebrafish with depletion of *c9orf72* and expression of 30 CAG repeats had an abnormal motor behavior with reduced touch-evoked escape response. This also disrupted arborization and caused shortening of motor neuron axons. Knock-down of *c9orf72* alone did not produce any locomotor phenotype, spinal motor neuron aberrations, polyQ aggregation or toxicity. Combination of knock-down of *c9orf72* with *atxn2* with normal polyglutamine length (22 repeats) did not produce these aggregates and neuronal cell death. Additionally, the intermediate expansion of 30 repeats of CAG alone did not produce toxicity in zebrafish. This suggests that intermediate polyglutamine expansions in *atxn2* might be a genetic modifier of ALS-FTD [48,49]. This is not a real model of SCA2, because they only used intermediate expansions of *atxn2* in combination of knock-down of *c9orf72*. However, it is interesting that these studies found aggregation of intermediate repeats of *atxn2* that seem to be a result of dysregulation of the autophagy produced by *c9of72* reduction. Future generations of zebrafish models with pathological expansions of more than 35 *atxn2* repeats could reproduce the *atxn2* aggregation and toxicity alone without *c9of72* knock-down.

#### 3.1.2. SCA3

Spinocerebellar ataxia type 3 (SCA3), also known as Machado–Joseph disease, is a polyglutamine (polyQ) neurodegenerative disorder caused by abnormal (more than 40 repeats) CAG nucleotide repeat expansions in the ataxin-3 (*ATXN3*) gene, which encodes a protein that is involved in ubiquitin-proteasome system degradation of proteins [27,28].

The first SCA3 zebrafish model was made by Liu and colleagues [50] by injecting 80 polyQ repeats mRNA in zebrafish embryos and showing that this caused apoptosis, mainly in the CNS, at early developmental age. When they injected the *atxn3* repeats in a knock-out model of *p53*, neuronal degeneration was not observed. As P53 has known functions in cycle arrest and apoptosis, this indicates that expression of *atxn3* polyQ repeats induces selective transcription/expression of *p53* target genes and promotes *p53*-dependent apoptosis in the CNS of zebrafish [50]. 

Maxinne Watchon and colleagues [51] generated the first transgenic SCA3 zebrafish through the injection of 23 and 84 polyQ repeats. *Atxn3*-84Q zebrafish showed decreased survival compared to *atxn3*-23Q and developed neuropathy with polyglutamine neuritic beading-staining pattern in the medulla, *atxn3* cleaved fragments, and motor impairment that resulted in slower swimming. Potential drugs for treatment of SCA3 were found using this mutant zebrafish. Calpeptin (a calpain inhibitor) decreased levels of *atxn3* cleaved fragments in *atxn3*-84Q zebrafish and rescued the motor phenotype, but it also removed all ATXN3 expanded protein due to an increase in autophagic flux (indicated by reduced *p62* levels and increased LC3II levels) that cleared autolysosomes. Cotreatment with the autophagy flux inhibitor chloroquine prevented the removal of human ATXN3 protein and improved swimming [51]. Surprisingly, they did not study the cerebellum of transgenic SCA3 zebrafish, and they only tested zebrafish of 1-year-old (i.e., middle aged) fishes, and not older individuals in which neurodegeneration could be higher.

These results demonstrate the toxicity of *atxn3* polyQ repeats, show the possible relevance of calpeptin in the treatment of SCA3 and highlight the value of zebrafish as a model to test drugs in expansion disorders.

More recently, an *atxn3* morphant model resulted into small eyes with defective retinal structure and disorganization of the microtubule cytoskeleton structure. The morphant embryos that had a less severe phenotype had an alteration of the length of the outer segment of photoreceptors and opsin mislocalization. Coinjection of human mRNA rescued the phenotype but the coinjection of human mRNA with 14 or 80 repeats did not rescue completely the phenotype. These data suggest a role for *atxn3* in retinal ciliogenesis and phagocytosis [52]. This could explain some cases of retinal alterations found in SCA3 patients [53,54,55].

#### 3.1.3. SCA6 and Episodic Ataxia 2

Spinocerebellar ataxia type 6 (SCA6) is caused by mutations leading to CAG repeats (20 or more) in the *CACNA1A* gene that encodes the α_1A_ voltage-dependent calcium channel subunit [29]. These channels are abundant in presynaptic terminals, mainly in Purkinje cells of the cerebellum where they control neurotransmitter release [32]. In addition, missense mutations in the *CACNA1A* gene are also associated with episodic ataxia type 2 and hemiplegic migraine [43,44,45].

In zebrafish, the *CACNA1A* gene is duplicated into the *cacna1aa* and *cacna1ab* genes. A knock-out model for *cacna1ab* showed progressive loss of touch-evoked motor behaviors that was not explained by the lack of elements of touch-evoked circuit. *Cacna1ab* mutant embryos had Rohon-Beard neurons and Mauthner cells with their normal projections. However, touch-evoked activation was absent in *cacn1ab* mutant homozygous. Furthermore, heterozygous fish displayed an intermediate behavioral phenotype and the injection of a splice blocking morpholino reproduced the homozygous phenotype [56]. A knock-out model for *cacna1ab* showed reduced motility with weak and brief swimming bouts caused by a defect in neuromuscular transmission in homozygous fishes. These defects in neuromuscular transmission are explained by reduced calcium trafficking at the presynaptic neuromuscular junctions. Treatment with 3,4-diaminopyridine (a K^+^ channel blocker) and roscovitine (a P/Q-type channel agonist) rescued locomotion and neuromuscular transmission [57].

Morpholinos for *cacna1aa* caused reduced locomotor activity, behavioral impairment and epileptic-like seizures. *Cacna1aa* expression was detected in optic tectum and medulla oblongata at 4 days post-fertilization (dpf) larvae. Larval *cacna1aa* knock-down increased fish mortality probably due to defects in the brain, in the periphery, or both. *Cacna1aa* morphants had remarkable morphological malformations: curved body axis, reduced head and eye size, pericardial edema and yolk sac malformations. Peripheral effects of loss of function of *cacna1aa* were evidenced by slight hyperpigmentation, lack of swim bladder and shorter body length. Interestingly, these phenotypes have been also observed in other zebrafish models of epilepsy [58,59]. Moreover, knock-down of *cacna1aa* induced epileptiform-like effects in 90% of larval zebrafish. When antiseizure drugs were assessed (sodium valproate, ethosuximide, lamotrigin and topiramate), the epileptiform-like events of 4 dpf *cacna1aa* morphant larvae were significantly diminished [60].

Neural damage was observed in SCA6 zebrafish models. However, as SCA6 is a dominant hereditary ataxia, more efforts in the study of heterozygous models such as that developed by [56] or the creation of models of CAG repeats, will be necessary. However, as point mutations are also associated with episodic ataxia 2, which sometimes is presented with epilepsy [61], these models would help to learn more about molecular mechanisms that cause loss of function of *CACNA1A* gene.

#### 3.1.4. SCA7

Spinocerebellar ataxia type 7 (SCA7) is characterized by progressive neuronal loss in the cerebellum and associated structures and loss of visual acuity due to loss of rod-cone photoreceptors [30,31,32,33]. It is produced by CAG repeats in the N-terminal region of ataxin-7 gene (*ATXN7*), which is a subunit of a multiprotein complex, the Spt-Ada- Gcn5-acetyltransferase (SAGA) complex, that is involved in histone acetylation and transcription regulation [62].

In zebrafish, morpholinos for *atxn7* caused an increased embryonic lethality (most of them died before 24hpf) combined with severe developmental defects like impaired head and tail differentiation. Moreover, a small amount of morpholino was enough to cause a marked disorganization of the photoreceptor layer and reduction of the number of photoreceptors in the retina. In addition, *atxn7* morpholinos impaired Purkinje and granule cell differentiation. TUNEL assay at 5 dpf in *atxn7* morphants revealed that the reduced number of cerebellar neurons was not caused by increased levels of apoptosis. The phenotype was rescued by coinjection of human ATXN7 mRNA [63].

Carrillo-Rosas and colleagues [64] inactivated the *atxn7* gene with morpholinos and CRISPR Cas9 and both models developed coloboma that was rescued by human *ATXN7* mRNA coinjection. *Atxn7* predominant expression in the ocular area already at 18 hours post-fertilization (hpf) suggested extensive functions during eye development. Moreover, dysfunction of *atxn7* led to an increase in sonic hedgehog signaling and alteration of proximo-distal patterning of the optic vesicle. Optic nerve formation was altered through affectation of ganglion cell axon pathfinding and optic nerve bundling, which is consistent with increased sonic hedgehog signaling. Photoreceptor terminal differentiation was also altered due reduced expression of cone-rod homeobox protein [64].

The retina of zebrafish has been studied for a long time, and many mechanisms involved in its development and function are known [65,66]. Therefore, these models offer an easy and accessible way to study the function of *atxn7* in neural and eye development in zebrafish. In the future, it would be of interest to generate zebrafish models with the CAG repeats to mimic better the genetics of SCA7.

#### 3.1.5. SCA13

Spinocerebellar ataxia type 13 (SCA13) is caused by missense mutations in the *KCNC3* gene, which encodes the voltage-gated potassium channel Kv3.3 [34,35]. Kv3.3 channels are highly expressed in Purkinje cells [67].

Zebrafish Kv3.3 channels exhibit strong functional and structural homology with mammalian Kv3.3 channels. Human SCA13 mutations had similar effects on the activity of zebrafish Kv3.3 channels [68]. The first zebrafish model of SCA13 was made by the injection of human *KCNC3* mRNA with a dominant negative R420H late onset subunit. Expression of this mutation significantly suppressed the excitability of Kv3.3 expressing, fast-spiking neurons in zebrafish. Although there were no gross locomotor deficits, precision and amplitude of the startle response was significantly reduced at 55-60 hpf. These data support the idea that changes in neuronal excitability initiate pathogenesis in SCA13 [69].

Differences in neuronal development in early or late onset SCA13 was studied with the expression of infant or adult-onset mutant proteins in motor neurons in the zebrafish spinal cord. Early onset human mutation (F448L) expressed in zebrafish caused recurrent pathfinding failure of the caudal primary motor neurons, which sent long abnormal collateral axons to inappropriate territories in the musculature. However, adult-onset human mutation (R420H) in zebrafish did not produced pathfinding errors but contributed to extend the complexity of the distal axonal arbor. These results seem to indicate that early onset SCA13 is related with notable changes in the development of cerebellar neurons that express Kv3.3 channels, which may contribute to the severe cerebellar atrophy seen in affected infants [70].

A SCA13 zebrafish transgenic line coding an adult onset (R420H) human mutant kv3.3 channel with specific expression in cerebellar Purkinje cells derived into a strong degenerative phenotype linked to extensive Purkinje cell degeneration of their dendritic and axonal structures. Movement deficits, as observed in SCA13 patients, were also characterized by a significant reduction in numbers of saccade eye movements [71].

Differential excitability and viability of Purkinje cells in early onset and late onset mutations was proved using zebrafish transgenic lines expressing infant onset or adult-onset mutations in Purkinje cells. Zebrafish Purkinje cells expressing an early onset mutation suffer a transient hyperexcitability shortly after Purkinje cells become spontaneously active. This hyperexcitability stops the extension of Purkinje cell processes, altered dendritic branching and synaptogenesis and resulted into cell death during cerebellar development. This effect of infant-onset mutation reproduced the aberrant development and atrophy of the cerebellum in early onset SCA13 patients. However, zebrafish Purkinje cells expressing adult-onset mutation, matured normally and survived during cerebellar development. Only a latent reduction in excitability was observed after an interval of evoked, high frequency spiking. This resembles the phenotype of SCA13 adult-onset patients in which cerebellar degeneration starts in adulthood [72]. The differences in Purkinje cell phenotype of adult-onset mutation between this study, [72] and [71] could be explained by the expression of the mutant protein in [71], which might be the cause of Purkinje cell degeneration. Toxicity of the aberrant protein might be a more decisive cause of late onset SCA13 than latent Purkinje cell hypoexcitability.

These studies revealed the validness of the zebrafish model to study degeneration of motor neurons and Purkinje cells caused by SCA13 mutations and elucidate the function of Kv3.3 channels. Although zebrafish models reproduced differences in early and late onset SCA13 mutations in excitability, pathfinding and viability of Purkinje cells, it would be necessary to develop an adult zebrafish expressing late onset mutations to elucidate what are the process that affect adult-onset cerebellar degeneration.

#### 3.1.6. SCA14

Spinocerebellar ataxia type 14 (SCA14) [36] missense mutations in the *PRKCG* gene, which encodes the γ-isoform of protein kinase C (PKCγ), causes increased kinase activity, amyloid citotoxic aggregates and apoptosis via impairment of the ubiquitin proteasome system and induction of endoplasmic reticulum stress [73,74,75]. PKCs are involved in intracellular signaling in numerous cellular processes. PKCγ is mainly expressed in the CNS, being especially abundant in cerebellar Purkinje cells, and plays an important role in synaptogenesis [75].

Patten et al. [76] studied with morpholinos if activation of PKCγ is required for the developmental speeding of α-amino-3-hydroxyl-5-methyl-4-isoxazolepropionate receptor (AMPA-R) that is necessary for correct maturation of glutamate synapses. They tested the presence of PKCγ by recording miniature excitatory postsynaptic current (mEPSC) of AMPA-R in presence of the drug Phorbol 12-myristate 13-acetate (PMA) and/or 5 mm K+, which were seen to be capable of increasing AMPA mEPSC amplitude by activating endogenous PKCγ [77]. In PKCγ morphants there was no effect on mEPSC amplitude, demonstrating that PKCγ expression was reduced. When PKCγ wild-type mRNA was injected with the morpholino, AMPA mEPSC amplitude was rescued. PKCγ morphants failed to hatch out of the chorion and did not exhibit the C-start escape response. These suggest that PKCγ activity is crucial for the proper behavioral development of zebrafish. Additionally, there was no observed maturation of AMPA-Rs in the PKCγ morphants at 48hpf with respect to embryos at 33hpf. Increasing synaptic activity in 33hpf embryos by application of an elevated K+ concentration or by application of N-methyl-d-aspartate induces rapid PKCγ-dependent trafficking of fast AMPA-Rs to synapses. These results seem to indicate that PKCγ is required for the normal developmental switch from slow to fast AMPA receptors in embryonic zebrafish Mauthner cells and its deficiency causes a failure in hatching and escape response [76].

The aim of the study by Patten et al. [76] was focused on understanding the function of PKCγ on AMPA receptors. As far as we know, zebrafish models were not used for study SCA14. Moreover, the PKCy mutation that produces SCA14 is a missense mutation, the protein is formed but the mutation causes misfolding, aggregation and toxicity [75]. Therefore, knock-down with morpholinos may not be the best way to model the disease and, knowing the advantages of the zebrafish as a model (see introduction), a mutant model more representative of the human disorder would facilitate our understanding of PKCγ function and SCA14 pathogenesis.

#### 3.1.7. SCA17

Spinocerebellar ataxia type 17 (SCA17) is caused by CAG/CAA polyglutamine repeats in the TATA-binding protein (TBP). TBP is an important general transcription initiation factor and the DNA-binding subunit of RNA polymerase II transcription factor D [37].

In 2001, Müller and colleagues [78] studied the role of TBP and TLF (TBP-like factor) in zygotic transcription in zebrafish. A *Tlf* model was generated with a dominant-negative variant of *TLF* mRNA. Embryos injected with a dominant-negative variant of *TLF* mRNA failed to initiate epiboly or arrested before dome stage. Additionally, expression of the endogenous notail (ntl) transcription factor, which is characterized by a ring like expression pattern at early gastrula stage embryos, was abolished in *TLF* injected embryos. Coinjection of wild-type *tlf*, rescued the phenotype and also rescued *ntl* mRNA expression. *Tbp* morphant embryos developed impaired epiboly and the phenotype was partially rescued by the coinjection of mRNA of human *TBP*. However, *ntl* expression was not affected in *tbp* morphant embryos. This suggests that *tbp* is not universally required for all zygotic polymerase II transcription in the zebrafish embryo. Nevertheless, the similarities between the two models seem to indicate that these two genes are essential during embryogenesis. For this reason, the expression of seven early patterning genes (*ntl*, *forkhead4*, *spadetail*, *goosecoid*, *even-skipped1*, *sonic hedgehog* and *T-box6*) was analyzed in both models. *Tlf* blocked embryos abolished expression of all genes with the exception of even-skipped1, which encodes a transcriptional repressor. *Tbp* morpholinos abolished the expression of five of the seven genes excepting *ntl* and forkhead4. In conclusion, it was demonstrated that *tbp* and *tlf* are required for epiboly in zebrafish embryos; although, they have different functions [78].

A subsequent deeper analysis of the same *tbp* knock-down model studied the function and mechanisms of *tbp* in transcription and early embryo development [79]. For this, they analyzed using microarrays the expression of 1927 genes at the dome stage in *tbp* morphant embryos. 17.5% of these genes showed a significant reduction of expression and a similar percentage (17.1%) had a significant increase. Meta-analysis of the ontogenic stage-dependent gene expression array indicated that most of the genes that require Tbp for their activation (77%) were principally stage-dependent and genes with reduced expression in *tbp* morphants showed the opposite tendency. A total of 23 GFP constructs using promoters of zebrafish genes expressed at the sphere/dome-stage were tested to discern whether Tbp regulates steady-state levels of mRNA in zebrafish through transcriptional or post-transcriptional mechanisms. Seven promoters displayed Tbp-dependent promoter activation, which confirms the suggested function of Tbp in activation of zygotic transcription of some genes during development. Four promoters showed an increase of promoter activity upon loss of *tbp*, which suggests a negative regulatory role of Tbp on the *tbp* gene promoter and support previous observed inverse correlation between *tbp* mRNA and Tbp protein levels at the late blastula and early gastrula stages [80]. In addition, the contribution of *tbp* to maternal mRNA degradation was demonstrated by searching maternal expressed genes in the *tbp* morphant microarray gene sets compared with an independent set of 662 maternal mRNAs and with the quantification of the expression of some maternal transcripts in morphants and controls. Maternally inherited transcripts were significantly upregulated in *tbp* morphants, which could indicate that the upregulation might be due to the specific loss of degradation of many maternal mRNAs. The use of a synthetic maternal mRNA *smad2* injected in cell embryos, allowed to observe its fate. Microinjected *smad2* mRNA was more efficiently degraded in control embryos than in *tbp* morphants, which demonstrated that the increase of smad2 mRNA levels in *tbp* morphants is due to the loss of degradation of *smad2* mRNAs. Embryos treated with a-amanitin at a concentration that inhibits Pol II showed higher levels of maternal genes similarly to *tbp* morphants. Finally, miR-430 microRNA mediated maternal mRNA degradation was observed to be specifically affected in *tbp* morphants when microarray data was analyzed and miR-430 target genes showed upregulation in *tbp* morphants [79].

Although function of *tbp* in transcription and early embryonic development was demonstrated, the role in neurodegeneration was not tested in these zebrafish studies. The development of models that express CAG/CAA expansions would be useful for that purpose.

#### 3.1.8. SCA37

Spinocerebellar ataxia type 37 (SCA37) is caused by the pentanucleotide repeated insertion ATTTC between ATTTT/AAAAT repeats in the non-coding region of the reelin adaptor protein DAB1, which is necessary for brain patterning and synaptogenesis [38].

Injection of the pathological allele insertion (ATTTC)58 in zebrafish embryos resulted in significant cell death that was not be observed in the normal N(ATTTT)7 and N(ATTTT)139 alleles, suggesting an RNA-mediated toxicity mechanism [38].

The function of reelin, *DAB1* and *VLDLR* (very low-density lipoprotein receptor), which are part of reelin signaling pathway, was later studied using zebrafish knock-out mutants. Specifically, these studies analyzed the role of these genes in synaptic lamina formation [81], behavioral phenotype [82] and the formation of the cerebellum and cerebellum-like structures [83].

Reelin signaling pathway during synaptic lamina formation was studied using mutants of pathway members DAB1 and VLDLR. Loss of function of reelin signaling pathway members critically disrupted lamina targeting of retinal ganglion cells axons and periventricular neurites in the tectal neuropil. *Vldlr* expression was restricted to the ganglion cell layer of the retina. There are two paralogous of *DAB1* in zebrafish, of which *dab1a* expression was found in retinal ganglion cells and *dab1b* in amacrine cells. CRISPR/Cas9 knock-out of *dab1a* resulted in abnormal retinal ganglion cell lamina targeting and increased arbor thickness in comparison with wild-type, although the phenotype was less severe than in reelin and *vldlr* mutants. Periventricular neurons with lamination defects were found to be increased in reelin, *dab1a* and *vldlr* mutants compared with wild-type. Transplanted retinal ganglion cells from wild-type into reelin mutants suffer serious laminar targeting defects when they innervate the tectal neuropil, although ganglion cell axons from reelin mutants into wild-type targeted correctly the single lamina in the neuropil. However, transplanted retinal ganglion cell axons from wild-type into *dab1a* and *vldlr* mutants did not exhibit laminar targeting defects. By studying reelin signaling it was also observed that *reelin*, *dab1a* and *vldlr* zebrafish mutants increase the average thickness of retinal ganglion cell projections into deep layers compared to wild-type. Overexpression of *vldlr* caused a significant reduction of retinal ganglion cells into deep layers, an effect no observed when *vldlr* was overexpressed in reelin mutants, which means that this had to be due reelin signaling [81]. Interestingly, VLDLR mutations have been associated with recessive cerebellar hypoplasia characterized by cerebellar ataxia, mental retardation, strabismus, dysarthria and seizures [84].

As abnormal Reelin signaling has also been observed in psychiatric disorders like autism [85], schizophrenia [86,87], bipolar disorder [86], and Alzheimer’s disease [88], the behavior of mutants for *reelin*, *dab1a* and *vldlr* was analyzed [82]. Reelin protein was confirmed to be expressed in a restricted pattern in the adult zebrafish brain, including the cerebellum with the exception of Purkinje and molecular cell layer. When reelin mutants were put in a social preference tank they displayed a selective reduction in their preference for social novelty in absence of global changes to social interactions, in contrast with wild-type and heterozygous embryos. In open field test wild-type, heterozygous and homozygous mutants for reelin spent a similar amount of time in the side and center of the tank, indicating that reelin homozygous exploration, anxiety behavior and aggression altered. *Dab1a* and *vldlr* mutants instead, exhibit a different behavioral phenotype. *Dab1a* mutants were hyperactive and more aggressive. *Vldlr* mutants had more exploratory behavior and less anxious behavior without being hyperactive. The different phenotypes in *dab1a* and *vldlr* mutants in comparison with the *reelin* mutant indicates that social behavior in *reelin* mutants is not mediated by the canonical reelin signaling pathway. In addition, levels of 5-HT in the hindbrain were seen to be increased in Reelin mutants by HPLC. When levels of 5HT were reduced with buspirone (5HT-1A receptor agonist), the interaction of the wild-type with unfamiliar fish increased while the mutant phenotype was not rescue. This means that at least in part 5HT signaling is involved in the preference for social [82].

The role of reelin in the positioning of the cerebellum and of cerebellum-like structures was also studied with *reelin*, *dab1a* and *vldlr* zebrafish mutants. Although no abnormalities were observed in the development of Purkinje cells or in layer formation in these mutants at 5dpf, adult mutant fish showed atypical Purkinje cell positioning in the cerebellum. Additionally, the proportion of ectopic Purkinje cells was higher in these mutants than in wild-type, which suggests that *reelin*, *dab1a* and *vldlr* are required for proper migration of Purkinje cells but not for their differentiation. Abnormal afferent axonal projections of parallel fibers and climbing fibers in dendrite regions of the ectopic Purkinje cells were found in the three mutant fishes. Ectopic eurydendroid cells and Bergmann glial cells were observed in reelin mutants, suggesting that reelin is involved in positioning of both cells. Abnormalities in cerebellum-like structures of the mesencephalic tectum were observed as aberrant parallel fibers in deeper regions near the ectopic type I neurons. Purkinje cells were observed in reelin mutants where they extended one or multiple neurites in aberrant directions. At 30dpf, more Purkinje cells were found in the granular cell layer in comparison with wild-type. Reelin was found in the tectum, cerebellum, and crista cerebellaris in parallel fibers, suggesting that reelin is transported in parallel fibers. When parallel fibers were ablated with laser, the localization of reelin was perturbed in the crista cerebellaris, which means that parallel fibers are necessary for the distribution of reelin at least in the dorsal hindbrain [83].

The studies of the role of *reelin*, *dab1* and *vldlr* in synaptic lamina formation, behavioral phenotype and in the positioning of the cerebellum and cerebellum-like structures were important to determinate the function and mechanisms of these genes. However, to focus research on modeling SCA37, it would be necessary to analyze more deeply the zebrafish embryos with pathological ATTTC expansions and investigate the mechanism by which these expansions cause the observed aberrations.

### 3.2. Other Dominant Ataxias

#### 3.2.1. Autosomal Dominant Sensory Ataxia 1

A missense mutation in *RNF170*, a ubiquitin E3 ligase gene, has been related to autosomal dominant sensory ataxia 1, which was found in two families from Canada [39]. In addition, biallelic mutations in *RNF170* were reported to cause autosomal recessive hereditary spastic paraplegia [89].

Injection of *rnf170* morpholino in zebrafish caused abnormalities and cell death before 1dpf in a dose dependent manner. Injection of *RNF170* human mRNA with the mutation caused developmental abnormalities in 79% of the embryos while injection of wild-type *RNF170* mRNA caused anomalies only in 18% of the embryos. Coinjection of the two mRNAs resulted in an intermediate proportion of disturbed embryos, 45%. These results suggested that *RNF170* mutation has a gain of function toxic effect even in the presence of the endogenous gene function [39].

Wagner et al. [89] used morpholinos against *rnf170* which resulted in microphthalmia, microcephaly and loss of motility at 48hpf. Acetylated-tubulin immunostaining, revealed reduced neurogenesis in *rnf170* morphants, especially in the mid-hindbrain region, and reduced motor neuron axon staining in the myotome. Hematoxylin-eosin staining of transverse cranial sections of 4dpf larvae revealed loss of ventricular cavities in *rnf170* morphants. Touch-evoked motility assay revealed loss of movement. Interestingly, coinjection with wild-type human *RNF170* mRNA exacerbated the developmental phenotype. Overexpression of human wild-type *RNF170* mRNA in wild-type embryos as in Valdmanis et al. [39] caused a moderate to severe phenotype in 50% of the embryos characterized by truncation of the body axis and reduction in eye size, while injection of pathological variants related with autosomal recessive hereditary spastic paraplegia did not cause an abnormal phenotype [89].

Although these assays with *rnf170* morpholinos caused neuronal degeneration and loss of movement, they did not ideally reproduce the autosomal dominant sensory ataxia 1. Zebrafish mutant models should be developed to support the morpholino discoveries and to gain new knowledge about this illness.

#### 3.2.2. Episodic Ataxia 1

Episodic ataxia 1 is caused by missense, nonsense and splice site mutations in the *KCNA1* gene, which produces a potassium channelopathy. Short-lived attacks of ataxia and sometimes epilepsy characterize this disorder [40,41].

*Kcna1a* zebrafish morphants were used as a drug screening platform to search antiseizure therapies. Bioenergetics measurements revealed increase in basal respiration, total mitochondrial respiration and ATP-linked respiration in kcna1a morphants but not in control zebrafish of 3dpf. This increase of basal respiration was not due to effects on the apoptosis pathway as was demonstrated with the coinjection of *p53* morpholino. Moreover, *kcna1a* morphants displayed hyperactivity when they were breed in darkness and EEG abnormal activity consistent in repetitive high frequency, large-amplitude spikes. A total of 23 antiseizure drugs that block elevated metabolism have been screened using this model; 15 of these compounds effectively restored basal respiration, maximum respiratory capacity and total mitochondrial respiration in *kcna1a* morphants to wild-type levels. A total of 870 compounds have been screened for their ability to significantly reduce hyperactive swimming behaviors. Vorinostat, an histone deacetylase inhibitor, was found to be a candidate drug to treat epilepsy, as it ameliorated bioenergetics measurements and significantly reduced seizures [90].

This *Kcna1a* morphant model seemed to reproduce some of the characteristics of episodic ataxia 1, although it was used only to study the epileptic phenotype. A future characterization of the model to search for more features of episodic ataxia 1 would be necessary. Furthermore, an efficient screening of antiseizure drugs was established based on the increased metabolism and seizures of the *kcna1a* model. This enabled the discovery of a candidate compound that would be useful for treating epilepsy. However, the creation of a mutant model would be a better approach.

#### 3.2.3. Episodic Ataxia 5

Episodic ataxia 5 is an extremely rare early-onset disease whose symptoms are generalized epilepsy and hereditary episodic ataxia. Missense mutations in calcium-channel b4 subunit gene (*CACNB4*) cause episodic ataxia 5 [42].

Ebert et al. [91] generated knock-downs in the *cancb4a* and *cancb4b* zebrafish genes to analyze if Ca^2+^ channel function is required for epiboly. Although cell division occurred normally in morphant blastoderms beyond this stage both *cancb4a* and *cancb4b* morphants developed three distinct types of phenotypes. Morphants of the most severe phenotype (class I) failed to initiate blastoderm epiboly with a ring of dead cells near the blastoderm margin, which caused the death of the embryos. Class II morphants initiated epiboly, but progressed slowly, with occasional cell death at the blastoderm margin. Surviving class II morphants developed severe morphological defects with extensive cell death in CNS and all died at 24 hpf. Class III (less severe) embryos died with cardiac defects at 6dpf. Injection of human mRNA of *CACNB4* rescued the phenotypes. Surprisingly, using a human cRNA incapable of binding calcium channels restored epiboly and coexpression of the mutations in *xenopus* oocytes. This led to the conclusion that *cancb4a* and *cancb4b* functions in epiboly were independent of Ca^2+^ channel activity. As *cancb4a* and *cancb4b* were expressed in blastoderm and yolk, injection of morpholinos in the yolk at 1000 cell stage produced embryos with class I phenotypes but in lower proportions than when injected in one cell stage embryo injections. Labeling the yolk of morphant embryos with the fluorescent reagent Sytox Green showed that the yolk syncytial layer had abnormal disorganized nuclei at the sphere stage when morphant morphology appeared normal. Nocodazole treatment, which is a microtubule disruptor, induced similar defects into yolk syncytial layer nuclei that knock-down embryos, which suggests that morphant zebrafish had a failure in microtubule polymerization [91].

*Cancb4a* and *cancb4b* morphants exhibited a dramatic lethal phenotype that did not reproduce the symptoms related with episodic ataxia 5. However, they help to understand the functions of *cancb4a* and *cancb4b* in epiboly and understanding the underlying molecular/cellular processes is of critical importance for the development of novel therapeutic strategies. A zebrafish mutant model might reproduce better the epilepsy and ataxia characteristics of episodic ataxia 5.

### 3.3. X-Fragile and FXTAS

Mutations on *FMR1* can produce two syndromes, depending on the penetration and number of CGG repeats in this gene that suppress the expression of the protein. Full mutation alleles with more than 200 CGG repeats develop X-fragile syndrome [46]. Premutation alleles on *FMR1* with 55 to 200 CGG repeats, present more frequently in carrier males than in females, cause fragile-X tremor ataxia syndrome (FXTAS) [47].

Morpholino knock-down of *fmr1* in the zebrafish brain caused disorganization and morphological changes in the midbrain/hindbrain boundary. Anterior extension of the head seemed to be incomplete in some *fmr1* morphant embryos at 24hpf. At 48hpf, some morphants displayed expanded brain ventricles and pericardial edema. Using a ventral telencephalon/diencephalon marker (*dlx-2a*) and a midbrain/hindbrain boundary marker (*fgfr1*), an altered pattern of transcription of both markers could be observed in *fmr1* morphants. Furthermore, an occasional axial defect was revealed in *fmr1* morphants. Coinjection with *fmr1* mRNA, modified to lack *fmr1* morpholino target sites, rescued the phenotype. Neurite branching in Rohon–Beard neurons in *fmr1* morphants was observed to be increased (2-fold). Moreover, an 8-fold increase in rare axon guidance defects was also reported. Neurite branching was also observed in axons of the trigeminal ganglion neurons. Treatment with MPEP, a metabotropic glutamate receptor (mGluR) antagonist, rescued the branching effects. Interestingly, treatment with MPEP in wild-type embryos resulted in significant simplification of neurite branching and reduction in branch termination. These data suggest that Fmr1 function influences neurite morphogenesis by facilitating mGluR signaling. Craniofacial anomalies were examined using Alcian blue staining. Meckels’ cartilage was significantly shorter and wider in *fmr1* morphants in comparison with wild-type. Treatment of *fmr1* morphants with MPEP returns the length and width of Meckels’ cartilage close to normal. In addition, reduced level of innervation of the facial region by the cranial nerves was also observed in *fmr1* morphants. As cranial ganglia and Meckels’ cartilage share a common origin in the neural crest, this suggests that these defects might be due to defective neural crest specification, migration or differentiation. Examination of neural crest-derived structures revealed significantly reduction of trigeminal ganglion neurons in *fmr1* morphants compared to wild-type larvae. However, MPEP treatment and *fmr1* mRNA injected embryos had increased number of trigeminal neurons in the ganglion than wild-type larvae. Defects in neural crest specification were found in *fmr1* morphants at 26-somite stage and migration of neural crest cells was found to be different in *fmr1* morphants than in wild-type. These data suggest that *fmr1* has a key role in specification and migration of a subset of neural crest cells [92].

In contrast to the morpholino study, two knock-out *fmr1* models created with TILLING were viable, developed into fertile adults, and did not display any phenotype. No craniofacial defects or Rohon-Beard neurite branching defects were observed [93]. Behavioral analysis in one of these two *fmr1* knock-out zebrafish lines revealed anxiolytic responses in *fmr1* mutants which spend significant more time in white compartment than wild-type and increased locomotion upon a light/dark test. Inhibitory avoidance test showed impaired inhibitory avoidance in *fmr1* mutants. Moreover, speed and distances moved were higher in *fmr1* knock-out fishes than in wild-type in an open field test, which demonstrated the presence of hyperactivity. Electrophysiological recordings from telencephalic slice preparations of adult fishes exposed significant reduction in long-term potentiation and enhanced long-term depression in *fmr1* mutants compared with wild-type fish, which suggested that *fmr1* mediates telencephalic synaptic plasticity [94]. Subsequent research on adaptation of *fmr1* adult mutants to a novelty environment over a twenty-minute period in open field test, revealed reduced neophobic responses. *Fmr1* mutants travelled longer distances in the first 10 minutes of open field test (considered time of habituation of a novel environment) than wild-type fishes, but after this time only vertical activity increased. This increase in travel distances of *fmr1* mutants in the first 10 minutes of the test was proposed to be due to reduced neophobia and not due to hyperactivity. In addition, analyzing turning behavior and movement patterning did not detect stereotypic behaviors. Moreover, *fmr1* mutants swam to deeper levels than wild-type in a tank with a white bottom, beyond the neophobic phase and preferred transparent walls. This was proposed to be a result of altered explorative behavior or reduced open space aversion instead of reduced white aversion [95]. A study of social behavior in *fmr1* mutants reported precocious development in shoaling preference at 28dpf in *fmr1* mutants. Light/dark test revealed elevated anxiety levels in *fmr1* mutants, which spent a higher percentage of time in the light zone at 14dpf and had a reduced number of midline crossings than wild-type. A novelty test showed that 14dpf zebrafish spent more time in the upper tank (which means reduced anxiety), but at 28hpf showed a decrease in time spent in the upper tank compared to wild-type [96]. Alteration of auditory processing in *fmr1* zebrafish mutants that were hypersensitive to sound was also reported [97]. *Fmr1* mutant larvae hypersensibility to sound could be a result of hyperactivity of the thalamus [97]. *Fmr1* patients also reveal enhanced evoked electrophysiological responses to sound [98].

Moreover, the function of *FMRP* in neural circuit formation was studied by Shamay-Ramot et al. [99] in the same model of *fmr1* knock-out. First, they tested if mRNA target genes *mTor* and *sash1,* which are related with *FMRP,* were affected and *fmr1* mutants were observed to have an increased expression of both genes. Locomotion was assessed into 6dpf *fmr1* knock-out larvae revealing hyperlocomotor activity under light and dark conditions and altered behavioral response to light stimuli. *Fmr1* mutant embryos of 2dpf had expanded length of the motor axon arbors of 59% and increased number of branches of 120% compared to wild-type embryos. Total synaptic density in the axons of spinal motor neurons increased by 53% in *fmr1* knock-out embryos. Rohon-Beard sensory neurons also had increased arbor length and number of branches of 73% and 92%, respectively, in *fmr1* mutants in comparison with wild-type. Synapse density was not affected in Rohon-Beard neurons of *fmr1* mutants. Glutamatergic neurons had a 30% increase in synapse density in *fmr1* mutants. Expression of Adar enzymes, which have functions in RNA editing, were measured in *fmr1* mutants. Co-immunoprecipitation of Fmrp and Adar2a proteins revealed biochemical interaction. RNA immunoprecipitation assays showed that Fmrp protein can bind *adar1* mRNA. Moreover, RT-qPCR of the four zebrafish *adar* genes revealed overexpression of all these genes in *fmr1* mutants. Western blot analysis of Adar2 protein showed a 30% increase in expression levels in *fmr1* mutant brains in comparison with wild-type brains. Analyses of RNA editing target sites revealed a mild increase in RNA editing of Adar target genes with synaptic and neuronal functions in *fmr1* mutant larvae of 6dpf. Tissue specific changes of RNA editing levels were observed when the same genes were analyzed in adult brains. These data suggest that Fmrp-mediated RNA editing plays a role in the plasticity of neuronal circuits [99]. Furthermore, a role of *fmr1* in myelin sheath development was demonstrated in the same *fmr1* mutant line in which its oligodendrocytes developed smaller myelin sheaths. Reduced myelin basic protein expression was also observed in *fmr1* mutant larvae [100].

Recently, *fmr1* mutant larvae created by CRISPR/Cas9 technology showed hyperactivity, learning and memory defects and impaired craniofacial cartilage development. In addition, genes related with memory function and cartilage development were found downregulated in *fmr1* mutants by RT-qPCR. Injection of wild-type *fmr1* mRNA rescued the phenotype [101].

A knock-down line created by DNAzyme reproduced some features of X-fragile such as structural deformities (craniofacial abnormalities, bent notochord and deformed tail) increased anxiety, impaired cognition and caused repetitive behavior (circling) at 7dpf. Moreover, mGluR5 protein, a direct target of FMRP increased. This model was used to find an accurate time window of treatment. Treatment in 0-3dpf window obtained the best results. Early treatment of the *fmr1* knock-down embryos with mavoglurant, an mGluR5 antagonist, reduced anxiety, increased cognition and reduced circling at 7dpf. Treatment with KU046 (an anxiolytic) in the 0-3dpf window also reduced anxiety and repetitive behavior at two doses [102].

These models revealed important functions of Fmrp, reproduced most of the clinical phenotypes and in consequence would be useful for high-throughput screening of different compounds. As was seen in [102], early pharmacological treatment would improve the effectiveness of the drugs that could be tested and maybe reduced disease progression. However, the creation of zebrafish models with CGG repeats would allow understanding the function of expansion repeats in X-fragile syndrome and FXTAS.

## 4. Conclusions

The majority of the zebrafish models reviewed here of genes related to dominant ataxia and X-fragile/FXTAS presented neural damage and/or locomotor deficits that recapitulate the human disorders. However, there are some models that did not develop any neuronal or locomotor deficit: *tbp* morphants [78,79] and *cancb4a* and *cancb4b* morphants [91]. Nonetheless, *tbp* models were made of morpholinos and SCA17 is a gain of function disorder but these studies helped to decipher *tbp* function in early development and gene transcription [78,79]. *Cancb4a* and *cancb4b* models were generated with morpholinos that revealed *cancb4a* and *cancb4* functions in epiboly [91], although heterozygous mutant models would have reproduced better the characteristics of episodic ataxia 5.

We should consider that most of the studies in zebrafish were performed with morpholinos, which can sometimes cause off-target effects. It is necessary to validate them with a control morpholino, mRNA rescue and Western blot/immunohistochemistry or RT-qPCR. Furthermore, occasionally comparisons of a morphant model with their respective mutant line revealed different phenotypes, which were usually milder in the case of mutant animals. This can be caused by genetic compensation and maternally mRNA rescue in the mutants. For that reason, is important and necessary to complement morpholino work with mutant studies [103].

Recently developed CRISPR/Cas9 technology makes it possible to expand the number of efficient specific zebrafish mutant lines to generate better models for human ataxias [20,104]. The study of heterozygous knock-out mutants would reproduce dominant ataxias caused by missense mutations. However, in the dominant diseases of repeat expansions that are caused by toxic gain of function mechanisms the creation of loss of function models by knock-down or knock-out might not be the best approach. On the other hand, loss of function models would make it possible to elucidate the function of the gene related to the disorder. In addition, recent studies indicate that in repeat expansion neurodegenerative syndromes a combination of gain of function and loss of function mechanisms might act synergistically [105,106]. This could explain the neurodegenerative phenotypes observed in most of the loss of function zebrafish models discussed here.

Knock-in models are more adequate for reproducing gain of function diseases. Nonetheless, the creation of knock-in models is difficult in zebrafish because it is necessary to trigger homologous directed repair, which is much less efficient in these animals [21]. New more effective knock-in techniques, such as the use of the CRISPR/Cpf1 system (instead of CRISPR/Cas9), which leads to a fourfold increase of knock-in efficiency [107], would help to establish the first knock-in zebrafish models of dominant ataxias.

Moreover, in expansion neuropathies, the injection of constructs with pathological repeats in zebrafish was found to be useful and revealed important data about toxicity and its mechanisms. For example, Huntington disease was broadly studied injecting pathological CAG expansions and these have allowed finding new possible treatments [108,109]. In this review, we described some zebrafish transient models of dominant ataxias made with the injection of pathological repeats that showed signs of neuropathy [38,48,50,51]. Moreover, the first transgenic model of dominant ataxia was created with the pathological expansions of SCA3 which had a locomotor deficit that was alleviated with chloroquine [51]. In the future, it would be important to expand the range of zebrafish models of pathological repeats for the study of different types of dominant ataxias. This could be applied to develop the first zebrafish models of other frequent dominant ataxias in which a similar zebrafish protein function was demonstrated, like SCA1 [110].

Zebrafish CNS organization allows the study of the cerebellum and its connections. However, there are some differences between human and zebrafish brains: absence of corticospinal and rubrospinal tracts in the zebrafish CNS [16], absence of the pons [111] and no direct telencephalic projections to the spinal cord [112]. Another disadvantage of zebrafish models is that 20 to 24% of zebrafish genes suffer a ray-finned fish whole genome duplication that generates genetic redundancy that could complicate model development [14]. Nonetheless, the advantages of zebrafish as a model for neuronal disorders are most relevant than these disadvantages. One of these main advantages is its utility as a model for high throughput screening of chemicals. In this review, we described some treatments that were applied successfully to zebrafish models of dominant ataxias [51,57,60,90,92] and X-fragile syndrome [102].

Finally, almost all research done with the zebrafish model in this field has been developed in embryos/larvae and there is a general lack of surveys in adult zebrafish models of late onset dominant ataxias. These would be more informative in the pathogenic functioning of the CNS because embryo/larvae models have not developed all neural connections. Furthermore, it could facilitate the search for treatments that target early pathological changes before the occurrence of toxicity and neuronal damage, which could reduce disease progression. The development of resources and techniques for zebrafish research will continue to enhance the utility of zebrafish for the study of human diseases and particularly in neurodegenerative diseases like ataxias.

## Figures and Tables

**Table 1:** Summary of molecular mechanisms and clinical features of the ataxias discussed in this article.

Disease	Gene	Mutation	Protein Function	Clinical Features	References
SCA2	*ATXN2*	35 to 59 CAG repeats	RNA binding protein that regulates mRNA stability	Progressive gait ataxia, dysarthria, Parkinsonian rigidity/bradykinesia, slow saccadic eye movements, tremor, muscle cramps, initial hyperreflexia followed by early hyporeflexia and myoclonus or fasciculation-like movements	[23,24,25,26]
SCA3	*ATXN3*	>40 CAG repeats	Involved in ubiquitin-proteasome system degradation of proteins	Slowly progressive ataxia accompanied with ophthalmoplegia, dysarthria, dysphagia, pyramidal signs, dystonia, rigidity and distal muscle atrophies	[27,28]
SCA6	*CACNA1A*	>20 CAG repeats	Control of neurotransmitter release	Slowly progressive cerebellar ataxia, dysarthria, dysarthria and mild vibratory and proprioceptive sensory loss	[29]
SCA7	*ATXN7*	37 to 200 CAG repeats	Involved in histone acetylation and transcription regulation	Cerebellar ataxia, dysarthria and dysphagia and loss of visual acuity	[30, 31, 32, 33]
SCA13	*KCNC3*	Missense mutations	Potassium channel activity	Early or late onset cerebellar ataxia with dysarthria often followed by mild intellectual disability and seizures	[34, 35]
SCA14	*PRKCG*	Missense mutations	Intracellular signaling in the Central Nervous System	Middle age of onset, slowly progressive cerebellar ataxia, dysarthria, nystagmus and myoclonus	[36]
SCA17	*TBP*	>43 CAG/CAA repeats	Transcription initiation factor binding DNA pol II	Ataxia, dementia and parkinsonism	[37]
SCA37	*DAB1*	>58 ATTTT repeats	Reelin adaptor, functions in neuronal development	Pure cerebellar ataxia and, distinctively, onset of dysarthria in late adolescence to adulthood	[38]
Sensory dominant ataxia 1	*RNF170*	Missense mutations	Ubiquitination	Severe loss of proprioception causing gait ataxia and a reduced ability to feel pain, temperature and vibration, particularly in the hands and feet	[39]
Episodic Ataxia 1	*KCNA1*	Missense, nonsense and splice site variants	Potassium channel	Early onset, spastic contractions of skeletal muscle, recurrent midline cerebellar dysfunction with loss of motor coordination and balance. Sometimes associated with epilepsy	[40, 41]
Episodic Ataxia 5	*CACNB4*	Missense mutations	Calcium channel	Early onset, generalized epilepsy and hereditary episodic ataxia	[42]
Episodic Ataxia 2	*CACNA1A*	Missense and nonsense mutations	Control neurotransmitter release	Ataxia episodic weakness, vertigo, dystonia, epilepsy nystagmus, and cognitive impairment	[43, 44, 45]
X-fragile	*FMR1*	X-fragile: >200 CGG repeats	RNA binding protein, Development of synapses	Mental retardation, facial dysmorphism, macroorchidism and autism like behavior or other psychiatric symptoms	[46]
FXTAS	*FMR1*	FXTAS: premutation alleles on FMR1 gene with 55 to 200 CGG repeats	RNA binding protein, Development of synapses	Late onset, cerebellar ataxia, intention tremor, dementia, parkinsonism and in sometimes psychological symptoms	[47]

## Data Availability

Not applicable.

## Supplementary Materials

The following are available online at https://www.mdpi.com/2073-4409/10/2/421/s1, Table S1: Description of zebrafish models of autosomal dominant ataxias cited in this article.
